# Assessment of interleukin-18 gene polymorphism and serum levels in cutaneous lichen planus

**DOI:** 10.1186/s40001-024-01846-z

**Published:** 2024-06-26

**Authors:** Reham William Doss, Abdel-Aziz El-Rifaie, Anton Nasr Roshdy, Dina Sabry

**Affiliations:** 1 Dermatology Department, Faculty of Medicine, Beni-Suef University, Mohammed Hassan Street, Beni Suef, 62511 Egypt; 2 Medical Biochemistry and Molecular Biology Department, Faculty of Medicine, Badr University in Cairo, Cairo, Egypt; 3 Medical Biochemistry and Molecular Biology Department, Faculty of Medicine, Cairo University, Cairo, Egypt

**Keywords:** Lichen planus, Autoinflammatory disorders, Cytokines

## Abstract

**Background:**

Lichen planus (LP) is a chronic inflammatory disease with uncertain etiology. Interleukin-18 (IL-18) is an interferon gamma (INFγ) inducing agent. It is a pro-inflammatory cytokine that was found to play a role in the pathogenesis of some autoimmune disorders.

**Material and methods:**

This study included 50 patients with classic cutaneous lichen planus (CLP) and 50 healthy volunteers serving as controls. Venous blood samples were withdrawn from the study subjects under complete aseptic precautions. Blood samples were examined for single nucleotide polymorphisms (SNPs) of IL-18 gene at promoter -137(G/C) and -656 (G/T) using polymerase chain reaction (PCR) and IL-18 level was assessed using enzyme linked immunosorbent assay (ELISA).

**Results:**

The mean level of IL-18 was significantly higher in CLP patients (31.63 ± 4.90) compared to control subjects (13.95 ± 6.82). Significantly high levels of IL-18 were found among patients with diabetes, hypertension (*p* < 0.01 in both). HCV positive patients and patients with both OLP and CLP also expressed higher levels of IL-18. Genotypic and allelic distribution at position -137(G/C) showed that the genotype GG was present at significantly higher frequency in cases (58%) compared to controls (28.0%). On the other hand the CC genotype at position -137 was significantly higher in the controls (28%) as compared to CLP cases (6%). Polymorphism of IL-18 at position -656(G/T) showed no significant difference between cases and controls. No significant difference could be detected in IL-18 level between different genotypic variants at position -137(G/C) and -656(G/T).

**Conclusion:**

IL-18 may play important role in pathogenesis of LP. Elevated IL-18 levels could be part of the pro-inflammatory autoimmune process in LP. The presence of OLP, HCV, diabetes and hypertension is associated with higher production of IL-18. IL-18 promotor region -137(G/C) polymorphism might be a factor that increase the risk of development of lichen planus in Egyptian patients.

## Introduction

The pathogenesis of LP is not entirely understood. In general, activated T lymphocytes (Both CD4^+^ and CD8^+^ T) recruited to the dermo–epidermal junction are responsible for apoptosis of basal keratinocytes [1].

The interaction between pathogenic T lymphocytes and basal keratinocytes is enhanced by increased expression of intercellular adhesion molecule-1 (ICAM-1) by basal keratinocytes, The up-regulation of the T-helper-1 (Th1) arm of cell-mediated immunity drives basal keratinocyte apoptosis. Implicated cytokines include interferon-γ (IFNγ) and tumor necrosis factor-α (TNF-α) [1].

IL- 18 belongs to IL-1 family of cytokines that is known to play a role in innate and adaptive immune responses; with the help of IL-2, IL-18 stimulates T helper-1 cells, NK cells, macrophages, dendritic cells and T cells to produce IFN-γ. IL-18 also acts in synergy with IL-3 to induce IL-4, IL-13 production from mast cells and basophils [2].

IL-18 has been found to play a role in the pathogenesis of inflammatory autoimmune skin diseases as psoriasis [3, 4]. Moreover Zhang et al. [5] reported significantly elevated levels of IL-18 in patients with oral lichen planus (OLP***).***

In this analysis we aimed to study the IL-18 serum level its genotypic variation in order to evaluate the role of IL-18 in the development of CLP in Egyptian population.

## Methods

This case-controlled study included 50 patients with CLP in addition to 50 healthy individuals serving as controls. Study subjects were consecutively recruited from the dermatology out-patient clinic at the Beni-Suef University hospitals in the period between January 2020 and January 2021.

Patients diagnosed with acute infection at time of sample withdrawal, those who were suffering from any autoimmune disease, skin disease other than LP or receiving any systemic drugs that might cause lichenoid reactions were excluded from the study.

### The following was carried out

1. The aim of our study was explained to each participant, the study was approved by the local research ethics committee of faculty of medicine, Beni-Suef university. A written informed consent for participation in the study was taken from each participant.
2. Full history including: age, gender, family history, distribution of the lesions, disease duration, previous treatments, recurrence, other medical conditions, history of drug intake was taken.
3. General medical examination was conducted to exclude any associated systemic disease.
4. Full dermatological examination to determine the type of LP and the surface area involved using visual estimation in which the area of the palm equals 1% of the total body.
5. Four weeks prior to sample withdrawal, patients stopped any systemic or local treatment for LP.

### Sample preparation

In sterilized EDTA tubes, serum was separated from 5 ml of venous blood by centrifugation. The separated serum was immediately stored at − 80 °C. Serum level of IL-18 was assessed using ELISA technique.

Genomic DNA was extracted from 400 μl venous blood samples and real time PCR reaction was conducted to detect IL-18 SNPs at position -137(G/C) and -656(G/T).

### Procedure

#### Enzyme-linked immunosorbent assay (ELISA)

Serum samples were collected in separator tubes and allowed to clot for 30 min before centrifugation at approximately 1000*g* for 5 min, and then kept at − 80 °C. Enzyme-linked immunosorbent assay (ELISA) assays were performed according to the manufactures’ instructions (R&D Systems Inc, Minneapolis, Minnesota, USA). The absorbance of the sample at 492 nm was measured with a spectrophotometer and the results were expressed in pg/ml.

#### Genotyping for IL18 gene polymorphisms

DNA was extracted using Omega DNA Blood Midi Kit (Omega Bio-Tek, Inc., Doraville, Georgia, USA). The SNPs at positions-137 (rs187238), -656 (rs1946519) in the human IL18 gene were analyzed by sequence-specific primers (SSPs) polymerase chain reaction (PCR) that uses SSPs with 3 end mismatches and identifies the presence of specific allelic variants through PCR amplification. All analyses were performed blindly with respect to the patient characteristics. The genomic DNA was measured using Nanodrop (Thermo Scientific, USA). We stored the samples at − 20 °C until forward usage. All primer sequences and fragment sizes are listed in (Table 1). The primers were all used at a final concentration of 10 μM. Genotyping was done using qPCR with Taman® allelic discrimination assay software (Applied Biosystems, Foster City, California, USA) using Applied Biosystems Step one qPCR System. The reaction was set with an input DNA of 20 ng and DNA amplification was done in a 25 µl volume which contains 12.5 µl TaqMan master mix, 1 µl forward primer, 1 µl reverse primer, 1 µl fluorescein (FAM) labeled probe, 1 µl 2′-chloro-7′phenyl-1,4-dichloro-6-carboxy-fluorescein (VIC) labeled probe, and the volume was completed with nuclease free water to 25 µl. Amplification was done under the following conditions: 95 °C for 10 min, then 35 cycles of 95 °C for 15 s, and then 60 °C for 1 min, and 60 °C for 30 s for annealing and extension. The primers and two labeled probes were determined through Real-Time TaqMan with assays (IL18 rs187238 and rs1946519) (www.appliedbiosystems.com). The passive reference dye used was 6-carboxy-X-rhodamine (ROX).

**Table 1:** Primer sequences and fragment sizes for IL-18 polymorphisms

Polymorphisms	Product size (bp)	Primer sequence
IL-18-137G/C	Consensus primer	5′-CAA TAG TCT GAA TGC AAA GCA GAT
-137G	798	5′-CCC CAA CTT TTA CGG AAG AAA AG
-137C	798	5′-CCC CAA CTT TTA CGG AAG AAA AC
IL-18-656G/T	Consensus primer	5′-TTC TGC ATC TTT ACA GCT GGA G
-656G	785	5′-TAA GCT TGG GGA GAG GGC
-656T	787	5′-AGT AAG CTT GGG GAG AGG GA

### Statistical analysis

The SPSS statistics software, version 26.0, 2018 was used to analyze the data (Tousand Oaks, CA, USA). Data was expressed as mean ± standard deviation. Categoric data was expressed as number and percentage. Chi-square test and Fisher’s exact test, and odds ratio (OR) and 95% confidence interval (CI) were used to assess the relative risk conferred by a particular allele and genotype. Hardy–Weinberg equation was used to assess allele frequency. For pairwise comparisons between the two groups, the independent *t* test was performed. One-Way ANOVA test was used for quantitative data between three groups. Statistical significance was assumed at the *p* < 0.05 level.

## Results

This study included 50 CLP patients and 50 healthy volunteers. The CLP patients included 20 male patients (40.0%) and 30 female patients (60.0%). Age of the patients ranged between 15 and 79 years with a mean (45.22 ± 15.42). The disease duration ranged from 1 to 240 months with a mean (47.96 ± 64.97).

The control group included 27 males (54.0%) and 23 females (46.0%). The age ranged between 15 and 72 years with a mean (34.90 ± 15.27). There was no significant difference between the patients and control groups regarding the demographic data.

22 patients (44%) had OLP lesions in addition to the CLP lesions, and, 15 patients (30%) were hepatitis C virus positive (HCV + ve).

The mean serum level of IL-18 was significantly higher in CLP patients (31.63 ± 4.90 pg/ml) compared to control subjects (13.95 ± 6.82 pg./ml) with (*p* value < 0.0001).

**Fig. 1:**
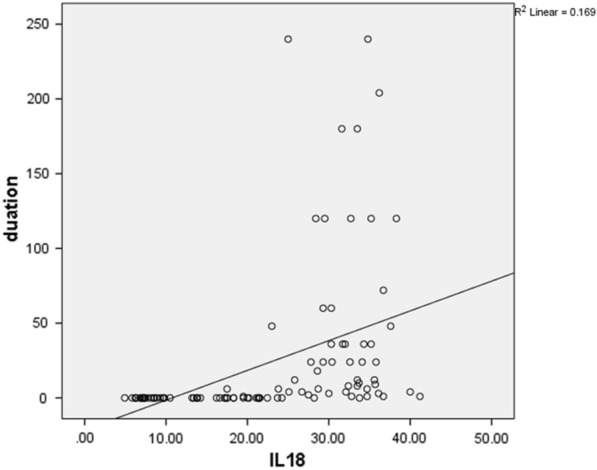
Correlation between the CLP disease duration and interleukin-18 level in serum

**Fig. 2:**
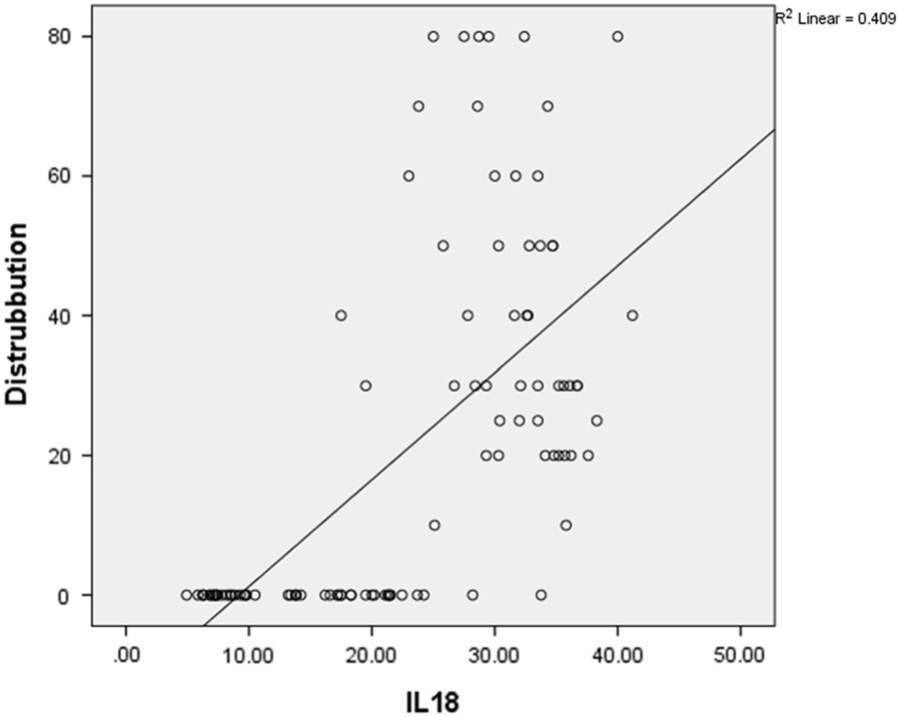
Correlation between the surface area of CLP and interleukin-18 level in serum

There was significantly positive correlation between the disease duration, The surface area involved and the level of IL–18 in serum [r = 0.411, *p* < 0.0001and r = 0.639, *p* < 0.0001 respectively) (Figs. 1, 2).

Significantly high levels of IL-18 was found among female patients, HCV + ve patients and patients suffering from diabetes mellitus (DM) and hypertension (Table 2).

**Table 2:** IL-18 level in serum according to demographic data

Characteristic	Number (%)	(pg/ml) mean ± SD	*p* value
Gender			
Male	20 (40%)	19.71 ± 10.44	0.0001*
Female	30 (60%)	29.02 ± 8.21	
OLP + CLP	22 (44%)	32.00 ± 4.74	0.0001*
CLP	28 (56%)	20.19 ± 10.45	
Erosive OLP + CLP	7	33.03 ± 3.82	0.50
Non-erosive OLP + CLP	15	31.53 ± 5.17	
HCV + ve	15 (30%)	32.13 ± 5.59	0.0001*
HCV −ve	35 (70%)	21.14 ± 10.52	
DM			
Yes	5 (10%)	33.62 ± 3.04	0.019*
No	45 (90%)	22.22 ± 10.63	
HTN			
Yes	7 (14%)	33.42 ± 5.27	0.006*
No	43 (86%)	21.98 ± 10.56	
Female patients			
OLP + CLP	14	32.04 ± 4.04	0.383
CLP	16	30.25 ± 5.86	
Female patients			
Erosive OLP	4	32.7 ± 3.1	0.706
Non-erosive OLP	10	31.78 ± 4.5	

*t* test for quantitative data between groups

*LP* lichen planus, *CLP* cutaneous lichen planus, *OLP* oral lichen planus, *HCV* Hepatitis C virus, *HTN* hypertension, *DM* diabetes mellitus

* Significant level at *p* value < 0.05

Patients with both OLP and CLP expressed non- significant high levels of IL-18 compared to those with CLP only. Similarly patients with erosive OLP had non significantly elevated IL-18 levels compared to those with non-erosive OLP (*p* > 0.01 in all).

Results of genotypic frequencies of IL-18 at position-137(G/C) showed that GG genotype was present in 58.0% of cases as compared to 28% of controls. On the other hand, GC and CC genotype of IL-18 at position-137 were higher in control group as compared to CLP patients’ group [in controls: GC 44.0%, CC 28.0% compared to GC 36.0% and CC 6.0% of cases].

G allele was significantly more frequent in cases (75%) and C allele was more frequent in controls (47%).

There was a significant difference in the genotype distribution between the patients with CLP and the control subjects at position-137 (*p* value < 0.05).

No statistical differences were observed for the genotype distribution frequency at position- 656(G/T) (Table 3).

**Table 3:** Comparison between cases and controls regarding IL-18 polymorphism at positions -137(G/C) and -656 (G/T)

Characteristic	Cases (n = 50)	Control (n = 50)	*p* value	Post hoc test (*p* value)
-137(G/C)				
CC	3 (6.0%)	14 (28.0%)	0.002*	0.003*
GC	18 (36.0%)	22 (44.0%)		0.414
GG	29 (58.0%)	14 (28.0%)		0.002*
G allele	75.5%	53%	0.0009*	0.0007*
C allele	24.5%	47%		0.001*
-656 (G/T)				
GG	20 (40.0%)	22 (44.0%)	0.901	
GT	24 (48.0%)	23 (46.0%)		
TT	6 (12.0%)	5 (10.0%)		
G allele	65.36%	68.38%	0.65	
T allele	34.64%	31.62%		

Chi square test (if less than 20% of cells have expected count less than 5) or Fisher’s Exact test (if more than 20% of cells have expected count less than 5) for qualitative data between groups

One-Way ANOVA test for quantitative data between the three groups

^*^Significant level at *p* value < 0.05

There was no statistically significant difference between the CC, GG, GC-137(G/C) and TT, GG, GT-656 (G/T) genotypes regarding the IL-18 serum levels (Table 4).

**Table 4:** Relation between serum IL-18 level and genotypic distribution for genotypes at position -137(G/C) and -656 (G/T) in cases and controls

Characteristic	IL-18 (pg/ml) Mean ± SD (Cases)	*p* value	IL-18 (pg/ml) Mean ± SD (Controls)	*p* value
-137(G/C)				
CC	35.53 ± 1.04	0.230	15.95 ± 5.25	0.405
GC	32.23 ± 4.32		13.57 ± 7.26	
GG	30.83 ± 5.30		12.56 ± 7.51	
-656 (G/T)				
GG	31.78 ± 4.88	0.887	13.34 ± 6.55	0.355
GT	31.32 ± 5.15		15.49 ± 7.26	
TT	32.36 ± 4.90		11.49 ± 6.26	

Chi square test (if less than 20% of cells have expected count less than 5) or Fisher’s Exact test (if more than 20% of cells have expected count less than 5) for qualitative data between groups

One-Way ANOVA test for quantitative data between the three groups

^*^Significant level at *P* value < 0.05

There is no difference in genotypic distribution at position-137(G/C), -656(G/T) in the cases group between male patients and female patients (*p* value > 0.05 in all) (Table 5, 6)**.**

**Table 5:** Gene polymorphism -137(G/C) relation to demographic data

Characteristic	CC	GC	GG	*p *value
Gender in cases				
Female	2 (6.7%)	9 (30%)	19 (63.3%)	0.56
Male	1 (4.5%)	9 (40.9%)	10 (45.5%)	
Female cases	2 (6.7%)	9 (30%)	19 (63.3%)	0.03*
Female controls	6 (26.1%)	10 (43.5%)	7 (30.4%)	
Post hoc test (*p* value)	0.0503	0.312	0.018*	
Male cases	1 (4.5%)	9 (40.9%)	10 (45.5%)	0.064
Male controls	8 (29.6%)	12 (44.4%)	7 (25.9%)	
OLP + CLP	2 (9.1%)	7 (31.8%)	13 (59.1%)	0.67
CLP	1 (3.6%)	11 (39.3%)	16 (57.14%)	
Erosive OLP	1 (14.3%)	3 (42.85%)	3 (42.85%)	0.717
Non erosive OLP	1 (6.7%)	4 (26.7%)	10 (66.7%)	
No OLP	1 (3.6%)	11 (39.3%)	16 (57.1%)	
Erosive OLP	1 (14.3%)	3 (42.85%)	3 (42.85%)	0.65
Non erosive OLP	1 (6.7%)	4 (26.7%)	10 (66.7%)	
HCV + ve	1 (6.7%)	5 (33.3%)	9 (60.0%)	0.288
HCV −ve	16 (18.8%)	35 (41.2%)	34 (40.0%)	
DM				
Yes	0 (0%)	5 (100%)	0 (0%)	0.019*
No	17 (17.9%)	35 (36.8%)	43 (45.3%)	
Post hoc test (*p* value)	00.299	0.005*	0.0046*	
HTN				0.230
Yes	0 (0%)	2 (28.6%)	5 (71.4%)	
No	17 (18.3%)	38 (40.9%)	38 (40.9%)	
Female patients				
CLP	1 (6.3%)	5 (31.3%)	10 (62.5)	0.985
CLP + OLP	1 (7.1%)	4 (28.6%)	9 (64.3%)	
Female patients				
Erosive OLP	1 (25%)	1 (25%)	2 (50%)	0.757
Non Erosive OLP	0 (0%)	3 (30%)	7 (70%)	
No OLP	1 (6.25%)	5 (31.25%)	10 (62.5%)	

Chi square test (if less than 20% of cells have expected count less than 5) or Fisher’s Exact test (if more than 20% of cells have expected count less than 5) for qualitative data between groups

* Significant level at *p* value < 0.05

**Table 6:** Gene polymorphism -656(G/T) relation to demographic data

Characteristic	GG	GT	TT	*p *value
Gender in cases				
Female	10 (33.3%)	15 (50%)	5 (16.7%)	0.32
Male	10 (50%)	9 (45%)	1 (5%)	
Female cases	10 (33.3%)	15 (50%)	5 (16.7%)	0.49
Female controls	11 (47.8%)	10 (43.4%)	2 (8.7%)	
Male cases	10 (50%)	9 (45%)	1 (5%)	0.69
Male controls	11 (40.7%)	13 (48.1%)	3 (11.1%)	
OLP + CLP	6 (27.3%)	13 (59.1%)	3 (13.6%)	0.261
CLP	14 (50%)	11 (39.3%)	3 (10.7%)	
Erosive OLP	1 (14.3%)	4 (57.1%)	2 (28.6%)	0.284
Non erosive OLP	5 (33.3%)	9 (60%)	1 (6.7%)	
No OLP	14 (50%)	11 (39.3%)	3 (10.7%)	
Erosive OLP	1 (14.3%)	4 (57.1%)	2 (28.6%)	0.313
Non erosive OLP	5 (33.3%)	9 (60%)	1 (6.7%)	
HCV + ve	5 (33.3%)	8 (53.3%)	2 (13.3%)	0.758
HCV −ve	37 (43.5%)	39 (45.9%)	9 (10.6%)	
DM				0.595
Yes	3 (60.0%)	2 (40.0%)	0 (0%)	
No	39 (41.1%)	45 (47.4%)	11 (11.6%)	
HTN				0.75
Yes	2 (28.6%)	4 (57.1%)	1 (14.3%)	
No	40 (43.0%)	43 (46.2%)	10 (10.8%)	
Female patients				0.672
CLP + OLP	4 (40%)	8 (53.3%)	2 (40%)	
CLP	6 (60%)	7 (46.7%)	3 (60%)	
Female patients				0.181
Non-erosive OLP	4 (40%)	6 (40%)	0 (0%)	
Erosive OLP	0 (0%)	2 (50%)	2 (50%)	
No OLP	6 (37.5%)	7 (43.7%)	3 (18.7%)	

Chi square test (if less than 20% of cells have expected count less than 5) or Fisher’s Exact test (if more than 20% of cells have expected count less than 5) for qualitative data between groups

Significant level at *p* value < 0.05

The GG Genotype at position-137(G/C), was present more frequently at female patients (63.3%) compared to female controls (30.4%) (*p* value < 0.05), while the CC genotype is more frequently presented in the female controls (26%) compared to female patients (6.7%) (*p* value = 0.503) (Table 5).

59.1% of patients with both CLP and OLP had the genotype-137 GG and similar percentage had GT genotype at position -656(G/T); No significant difference could be detected in allelic distribution at position -137(G/C) and position-656(G/C) regarding presence of OLP, type of OLP or HCV (Tables 5, 6).

The Standardized Coefficients Beta (95% CIs) in linear regression model for factors associated with interleukin-18 level in serum showed that the gender as Beta (95% CIs) was 0.156 (0.008–7.054) and (-137 G/C) as Beta (95% CIs) was −0.139 (−3.961 to −0.079) were significant predictors for IL-18 but other factors were insignificant predictors to IL-18 level in serum.

## Discussion

Lichen planus is an inflammatory disease with autoimmune pathogenesis. Various pro-inflammatory cytokines such as IFN-γ, TNF-α, IFNα, IFN induced protein MxA were found to be involved in LP pathogenesis [6].

In this study, the mean serum IL-18 was screened using ELISA; CLP cases expressed higher levels compared to the control group; The level of IL-18 was found to be positively correlated with the disease surface area and duration which means that the higher the surface area involved the more the level of IL-18. Furthermore, Patients with combined cutaneous and oral lesions had higher level of IL-18 compared with those with only cutaneous lesions.

Previous studies evaluated the level of IL-18 in OLP. Zhang and his coworkers [5] reported significantly high mean serum and salivary IL-18 levels in OLP cases in an ethnic Chinese population. Serum IL-18 levels were significantly higher in the erosive OLP compared to non-erosive OLP. Abdel Hay et al. [7], evaluated the IL-18 in 72 Egyptian OLP cases and detected elevated serum levels of IL-18 they also reported elevated IL-18 in tissue of erosive OLP lesions compared to non-erosive lesions.

In our study, patients with both OLP and CLP especially those with erosive OLP showed non-significant higher IL-18 levels compared to those with non-erosive lesions. The non-significant results could be explained by the low number of cases.

The association between LP and HCV is well established through previous reports. HCV was found to induce LP through the production of IFN and IFN induced proteins [8]. In this study HCV + ve patients showed significantly higher levels of IL-18 serum levels compared to HCV−ve patients. No difference in allelic variation could be detected for position -137(G/C) and -605(G/T) regarding HCV positivity in the studied cases.

IL-18 may play a disease-promoting, pro-inflammatory mediator in LP. It is possible that LP pathogenesis may first involve IL-18-promoting Th1 cell activation, which augments Th1 cytokine such as TNF-α and IFN-γ production, thereby triggering keratinocyte apoptosis [5].

We detected higher level of IL-18 among patients with diabetes and hypertension. As previously reported by Sedimbi et al. [9] diabetic patients had higher levels of IL-18. The significantly high level of IL-18 detected in our patients could be related to diabetes itself or lichen planus.

Single-nucleotide polymorphism (SNP) is one of the most common forms of genetic variations that affect the human genome and mediate the individual susceptibility to some diseases. Over the last years it has become increasingly clear that individual genetic variations (SNPs) is an essential component of overall immune response that control susceptibility, prognosis and outcome of autoimmune disease.

Over the past years, studies and metanalyses aimed at mapping the role of genetic variation in the development of LP were conducted. Evaluation of the associations of SNPs in cytokines genes (TNF-α (308G/A), IL-6 (174G/C), IL-10 (592C/A, 819C/T, 1082A/G)) with OLP susceptibility have been previously addressed. The results were usually inconsistent and inconclusive due to the differences in ethnicity [10, 11].

The gene for IL-18 is located at the chromosome 11q22.2-22.3 and its promoter region is relatively unique as it contains multiple transcription initiation sites. Three SNPs were identified in the promoter of the IL-18 gene at positions-137G/C, -607C/A, and -656G/T, relative to the transcriptional start site [12, 13].

In this study, there was a statistically significant difference in the genotypic and allelic distribution between cases with CLP and the control subjects at position-137(G/C), the genotype GG and the G allele were present at a significantly higher frequency in the cases compared with controls. On the other hand, CC genotype at position-137(G/C) was more frequent in the control group. No statistical differences were observed for the genotype distributions frequencies at position-656(G/T). This is the first study done so far to address the IL-18 polymorphism in cutaneous lichen planus.

Previous studies on IL-18 -137G/C or -607C/A polymorphism in OLP were conducted. As for -137G/C polymorphism, a significantly increased risk of OLP susceptibility was found in a meta-analysis study by Liu et al. [11] In another study by Negi et al. [14] on Indian population, genotypic and allelic frequencies at position -137(G/C) showed that GG genotype and allele G were significantly higher in OLP cases, whereas, GC genotype and C allele were higher in the control group. Whereas polymorphism of IL-18 at position -607(C/A) showed no significant differences between cases and controls. Bai and his coworkers [15], could also identify a significant difference in the genotype distribution between the OLP cases and controls at position-607(C/A) in Chinese patients, and the genotype CC was present at a significantly higher frequency in the cases compared to those in the controls.

SNPs of the promoter of IL18 gene at position-137(G/C) was predicted to disturb the nuclear factor binding sites for the cAMP responsive element binding protein. Furthermore, it was found that SNPs in the promoter of IL-18 gene at the position -137(G/C) could influence the expression of IL-18 and IFNγ [16, 17].

In this study, no difference in IL-18 levels could be detected between different genotypes at position -137(G/C) and -656(G/T) in LP patients. This is the first analysis that evaluated the relationship between SNP in IL-18 gene promoter at position-137 (G/C) and -656(C/A) and serum IL-18 levels in cutaneous LP.

Regarding patients with concomitant OLP, we could not detect significant differences in IL-18 level between different genotypes at positions -137(G/C) and -656(G/T). Similarly, Negi et al. [14], could not detect any association of genotypic distribution at position-137 (G/C) and -607 (C/A) with serum IL-18 levels in OLP patients. On the contrary, Bai et al. [15] reported that polymorphism at the promoter region (-137G/C) is likely to exert a positive effect on IL-18 production in OLP patients.

The non-significant results in the current study could be explained by the low number of cases. Another explanation is that the IL-18 levels could not be influences solely by the IL-18 gene polymorphism, other factors could be involved waiting to be revealed.

## Conclusion

These results indicate that IL-18 may play important role in pathogenesis of LP. Elevated IL-18 levels could be related to the pro-inflammatory autoimmune process in LP as well as the associated OLP, HCV, diabetes and hypertension.

IL-18 promotor region -137(G/C) polymorphism might be a factor that increase the risk of development of lichen planus in Egyptian patients and this is unlikely with the variation at -656 (G/T) genotype. It remains to be elucidated whether the role of this polymorphism is also applicable in other ethnic groups.

These findings could pave the way for novel therapeutic approaches.

### Study limitation

The study was limited by the low number of cases and. Further well-designed studies with larger sample size on different ethnic groups are still needed to consolidate the study findings.

### What’s already known about the topic?

- Lichen planus is a chronic inflammatory disease with uncertain etiology.
- IL-18 is an interferon gamma inducing agent.

- IL-18 plays a role in the pathogenesis of some autoimmune diseases as psoriasis and oral lichen planus.

### What does this study add?

- High serum level of IL-18 could play a role in the pathogenesis of cutaneous lichen planus.
- IL-18 may play a disease-promoting, pro-inflammatory mediator enhancing the disease progression and severity
- SNPs of IL-18 gene at promoter -137(G/C) seems to be associated with genetic susceptibility to CLP

## Data Availability

Not applicable.
